# Retinoic Acid Promotes the Generation of Pancreatic Endocrine Progenitor Cells and Their Further Differentiation into β-Cells

**DOI:** 10.1371/journal.pone.0002841

**Published:** 2008-07-30

**Authors:** Maria Öström, Kelly A. Loffler, Sara Edfalk, Lars Selander, Ulf Dahl, Camillo Ricordi, Jongmin Jeon, Mayrin Correa-Medina, Juan Diez, Helena Edlund

**Affiliations:** 1 Umeå Centre for Molecular Medicine, Umeå University, Umeå, Sweden; 2 Diabetes Research Institute, University of Miami Leonard M. Miller School of Medicine, Miami, Florida, United States of America; Baylor College of Medicine, United States of America

## Abstract

The identification of secreted factors that can selectively stimulate the generation of insulin producing β-cells from stem and/or progenitor cells represent a significant step in the development of stem cell-based β-cell replacement therapy. By elucidating the molecular mechanisms that regulate the generation of β-cells during normal pancreatic development such putative factors may be identified. In the mouse, β-cells increase markedly in numbers from embryonic day (e) 14.5 and onwards, but the extra-cellular signal(s) that promotes the selective generation of β-cells at these stages remains to be identified. Here we show that the retinoic acid (RA) synthesizing enzyme *Raldh1* is expressed in developing mouse and human pancreas at stages when β-cells are generated. We also provide evidence that RA induces the generation of Ngn3^+^ endocrine progenitor cells and stimulates their further differentiation into β-cells by activating a program of cell differentiation that recapitulates the normal temporal program of β-cell differentiation.

## Introduction

The identification of secreted signal(s) that promotes the selective generation of β-cells from stem and/or progenitor cells represent a significant step in the development of stem cell-based β-cell replacement therapy for type 1 diabetes. Such signals may be identified by studying how β-cells are normally generated during pancreas development. Transcription factors with important roles during pancreatic development and endocrine cell differentiation have been identified, and Notch signaling has been shown control pancreatic cell differentiation by regulating the expression of the proendocrine gene *Neurogenin 3 (Ngn3)* [1]–[6]. In contrast, no secreted signal has been identified that selectively promotes the generation of β-cells during pancreas development.

Ngn3^+^ endocrine progenitor cells give rise to different endocrine cell types at different stages of mouse embryonic development [1], [2], [5]. Glucagon positive (Glu^+^) cells appear by embryonic day (e) ∼9.5 whereas insulin positive (Ins^+^) cells appear predominantly from ∼e14.5 and onwards. Between e14.5 and neonatal stages Ins^+^ cell numbers increase 4–10 fold more than other pancreatic endocrine cell types [7], [8]. Why Ngn3^+^ endocrine progenitor cells preferentially start to differentiate into Ins^+^ cells at ∼e14.5 remains unknown. One possibility is that a cell intrinsic program in Ngn3^+^ progenitor cells regulates the temporal order of generation of differentiated endocrine cell types. Alternatively, extra-cellular signals may induce distinct classes of Ngn3^+^ endocrine progenitor cells at different developmental stages.

The vertebrate ventral spinal cord is a well studied system with respect to the mechanisms by which different classes of progenitor cells and differentiated progeny, inter-neurons and motor neurons, are generated [9]–[11]. Pancreatic endocrine cells and neurons share many common features and in particular β-cells and spinal cord motor neurons show striking similarities in patterns of gene expression of several basic-helix-loop-helix (Bhlh) and homeodomain transcription factors [11]–[13]. The secreted signal retinoic acid (RA) has been shown to control multiple, sequential steps of motor neuron differentiation in the ventral spinal cord, including initial patterning of progenitors cells and specification of motor neuron subtype identity [11]. The similarities between β-cells and motor neurons suggest that RA may also control specification and/or differentiation of insulin-producing β-cells.

Here we show that RA receptor signalling is required in early pancreatic progenitor cells for pancreatic development. We also show that the RA-synthesizing enzyme Raldh1 is expressed at stages when β-cells are normally generated in embryonic mouse and human pancreas. Using an explant assay of pancreatic endocrine cell differentiation, we provide evidence that RA promotes both the generation of Ngn3^+^ endocrine progenitor cells and their further differentiation into β-cells. The identification of a secreted signal that induces the generation insulin producing β-cells will aid in the establishment of a stem cell-based therapy for type 1 diabetes.

## Results

### *Raldh1* expression coincides with β-cell differentiation in both mouse and human fetal pancreas

**Figure 1:**
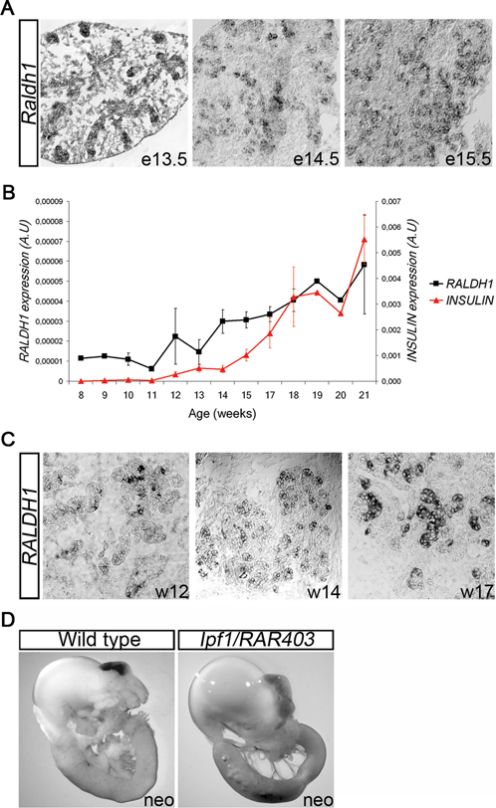
*Raldh1* expression in mouse and human embryonic pancreas. (A) *In situ* hybridization of an e13.5, e14.5 and e15.5 mouse pancreas using a *Raldh1* probe. (B) qRT-PCR using cDNA prepared from human fetal pancreas (n = 3 for week 8, 9, 10, 11, 13, 14 and 18, n = 2 for week 12, 15, 17 and 21, n = 1 for week 19 and 20) with *RALDH1* and *INSULIN* specific primers. (C) *In situ* hybridizations of week 12, week 14 and week 17 human fetal pancreas using a *RALDH1* probe. (D) The stomach-duodenal region from a wild-type and an *Ipf1/RAR403* transgenic neonatal mouse.

To address a potential role for RA in generation of endocrine progenitor and/or β-cells we first investigated expression of *Retinaldehyde dehydrogenase* (*Raldh)* genes in the developing mouse pancreas by *in situ* hybridization. Neither *Raldh 3* nor *4* was expressed in the developing pancreatic epithelium or mesenchyme at any stage examined (data not shown). Although *Raldh2* was expressed in e8.0 somitic mesoderm and e10.5 duodenal mesenchyme (Fig. S1a), it was not expressed within the developing pancreas. *Raldh1* was, however, expressed in the pancreatic epithelium from ∼e13.5 and onwards (Fig. 1a), coinciding with the period of massive β-cell differentiation. Expression of *Raldh1* in embryonic mouse pancreas was restricted to the branching ductal and acinar epithelium flanking the differentiating endocrine cells (Fig. S1b). qRT-PCR revealed that *RALDH1* expression overlapped temporally with that of *INSULIN* also in the developing human pancreas, whereas expression of *RALDH 2*, *3*, and *12* (the homolog of mouse *Raldh4*) was distinctly lower and/or barely detectable (Fig. 1b and data not shown). *In situ* hybridization analyses confirmed expression of *RALDH1* in fetal human pancreas (Fig. 1c). Thus, the expression of the RA-synthesizing enzyme Raldh1, coincides with the period of β-cell differentiation in both mouse and human pancreatic development.

### RA-signalling is required for pancreatic development

To address the role for RA-signalling in the developing pancreas we next generated transgenic (tg) mice expressing a dominant-negative (dn) form of the retinoic acid receptor (RAR) α, denoted *RAR403* [14], [15], under the control of the *Ipf1/Pdx1* promoter, which is active both in pancreatic progenitor cells and in β-cells [16]. The resulting *Ipf1/RAR403* tg mice showed complete pancreatic agenesis, lacking both dorsal and ventral pancreas, and died at the neonatal stage (Fig. 1d). These data are consistent with *Raldh2* expression in lateral plate and somitic mesoderm of e8.0 mouse embryos (Fig. S1a) and provides support for an intrinsic role for RA-signalling in specified Ipf1/Pdx1^+^ pancreatic progenitor cells. The perturbation of both dorsal and ventral pancreatic development in *Ipf1/RAR403* tg mice contrasts with the *Raldh2* ^−/−^ mutant mice in which dorsal but not ventral pancreatic development was affected [17], [18]. Taken together, our data provide evidence for a requirement for RA-signalling in early pancreatic progenitor cells of both the dorsal and ventral pancreatic anlage and suggest the existence of a *Raldh2* independent source of RA that signals to the ventral pancreatic anlage.

### Retinoic acid promotes the generation β-cells

The impaired pancreatic development in *Ipf1/RAR403* tg mice precluded any analysis of a role for RA-signalling in β-cell differentiation, and attempts to block RA signalling in β-cells by expressing the dnRARα in β-cells of tg mice using the *rat insulin 1* promoter^19^ failed to generate tg offspring, which may be due to non-pancreatic expression of the transgene at embryonic stages [20]. The elucidation of the roles of RA in motor neuron generation benefited largely from the use of an *ex vivo* explant system [9], [21]. Thus, to assess a potential role for RA signalling in β-cell generation we established an *ex vivo* explant system where e10.5 dorsal pancreatic buds were cultured in serum-free, defined media for six days in presence or absence of RA. Using these culture conditions the explants survive but do not grow.

**Figure 2:**
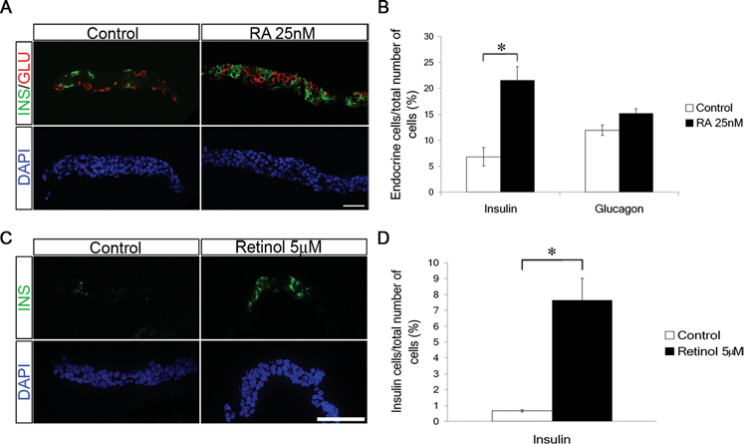
RA stimulates β-cell differentiation *in vitro*. (A) Representative sections of control and RA-treated e10.5 dorsal pancreatic explants cultured for 6 days and analyzed for insulin (green) and glucagon (red) expression. (B) Quantification of insulin and glucagon positive cells in control pancreatic explants and pancreatic explants exposed to RA for 6 days (n = 6 for both) displayed as % positive cells/total number of cells. (C) Representative sections of control and retinol-treated e10.5 pancreatic explants cultured for 6 days and stained with antibodies against insulin (green). (D) Quantification of insulin expression in control and retinol-treated pancreatic buds (n = 2 for both). Scale bar = 40 µM in (A) and 30 µM in (C). Data show mean±S.E.M, *p<0.05.

In explants cultured for six days in absence of RA, only a few glucagon positive (Glu^+^) and insulin positive (Ins^+^) cells were observed and the majority of cells were of non-endocrine identity (Fig. 2a,b and data not shown). In explants cultured in presence of 25nM RA a >3-fold increase in the relative number and ∼2,5 fold increase in absolute numbers of Ins^+^ cells (67±33 Ins^+^ cells in control explants and 155±23 Ins^+^ cells in RA treated explants, presented as mean±S.E.M) was observed, whereas there was no significant increase in the number of Glu^+^ cells (Fig. 2a,b and data not shown). Higher concentrations of RA (50 and 100 nM), failed to further stimulate the generation of Ins^+^ cells, and even showed an inhibitory effect on endocrine cell differentiation (data not shown).

The expression of *Raldh1* in the developing pancreatic epithelium (Fig. 1a–c) suggests that the epithelial cells themselves synthesize retinoids that participate in the induction of Ins^+^ cells. Previous studies have, however, shown that explants grown in medium are deprived of both RA and the metabolic substrate required for synthesis of RA by RA-synthesizing enzymes [21]. Thus, to test for RA-synthesizing activity in the pancreatic explants the RA precursor retinol was added to the medium. In explants cultured in presence of 5 µM retinol or 100 nM retinol a ∼10-fold increase in Ins^+^ cells was observed as compared to that of control explants (Fig. 2d and data not shown). Taken together, these results support the existence of an endogenous RA synthesizing system in the developing pancreas that promotes the generation of ins^+^ cells from pancreatic progenitor cells.

### Retinoic acid induces the expression of transcription factors that control endocrine cell differentiation

**Figure 3:**
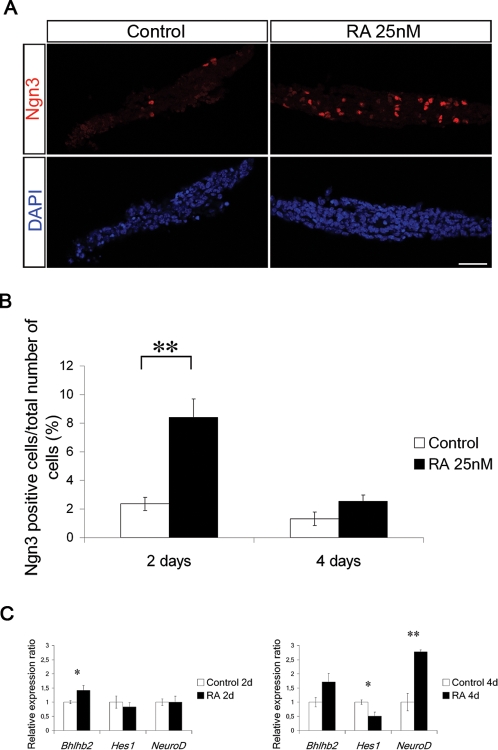
RA induces Ngn3 expression *in vitro*. (A) Representative sections of a control and RA-treated e10.5 dorsal pancreatic explants cultured for 2 days and stained with antibodies specific for Ngn3. (B) Quantification of Ngn3^+^ cells in control and RA-treated explants cultured for 2 or 4 days (n = 4 for each condition) displayed as % Ngn3 expressing cells/total number of cells. (C) qRT-PCR using *Bhlhb2*, *Hes1* and *NeuroD* specific primers on cDNA from control and RA treated e10.5 pancreatic explants cultured for 2 (n = 13) or 4 days (n = 9). Data represent the mean value±S.E.M. *p<0.05, **p<0.01, for RA treated explants versus controls.

In the ventral spinal cord RA induces a transcriptional program that culminates in the generation of a distinct set of motor neurons [9]–[11]. To begin to understand how RA promotes the generation of Ins^+^ cells in our explant system, we analyzed expression of transcription factors that regulate endocrine cell differentiation. Pancreatic endocrine cell differentiation, including that of β-cells, is preceded by and dependent on transient expression of the pro-endocrine gene *Ngn3* in progenitor cells [1]–[6]. Moreover, during motor neuron differentiation RA has been shown to regulate the expression of the pro-neural gene *Ngn2* [9]–[11]. Thus, we examined Ngn3 expression in explants harvested after 2 or 4 days of *in vitro* culture in presence or absence of RA. After 2 days of culture, explants exposed to RA showed a ∼4-fold increase in both relative (Fig. 3a,b) and absolute number of Ngn3^+^ cells (24±8 Ngn3^+^ cells in control explants and 94±24 Ngn3^+^ cells in RA treated explants, presented as mean±S.E.M) compared to controls. Consistent with the transient role for Ngn3 in pancreatic endocrine progenitor cell differentiation [1]–[6], the number of Ngn3^+^ cell was not significantly greater than that in control explants after an additional 2 days of RA exposure (Fig. 3b).

Ngn2 expression and thus motor neuron differentiation critically depends on the transient expression of Olig2, a Bhlh transcriptional repressor, induced by RA [9]–[11]. To elucidate whether expression of Olig2 or related Bhlh factors [22], [23] correlated with *Raldh1* expression in the pancreas we investigated expression of *Olig1-3,* and *Bhlhb2-5* in e13.5 and e15.5 pancreas by qRT-PCR. Only *Bhlhb2* was robustly expressed in the e13.5 and e15.5 pancreas (Fig. S2 and data not shown) and its expression was also increased by ∼50% in RA-treated explants at day 2 of culture (Fig. 3c). *NeuroD* is a Ngn3 target gene that is expressed in differentiated endocrine but not pro-endocrine cells. In agreement with the normal temporal order of expression of Ngn3 and *NeuroD,* no increase in *NeuroD* expression was observed at day 2 of RA exposure whereas an almost 3-fold increase in *NeuroD* expression was evident at day 4 of RA exposure (Fig. 3c). Thus, RA triggers the expected temporal sequence of events of transient Ngn3 expression followed by increased *NeuroD* expression.

During pancreatic and motor neuron development, activation of Notch results in increased expression of *Hes* genes that repress expression of the proendocrine gene *Ngn3* and the pro-neural gene *Ngn2*, respectively, thus maintaining cells as undifferentiated progenitor cells [12], [13]. *Bhlhb2* has been shown to inhibit Notch mediated activation of the *Hes1* promoter by interacting with the intracellular form of Notch [24]. In agreement with the increased expression of *Bhlhb2* in RA treated explants at day2, *Hes1* expression was reduced by RA to only ∼50% of that of untreated controls at day 4 (Fig. 3c). Thus, sequential stimulation of *Bhlhb2*, *Ngn3*, and *NeuroD* expression, paralleled by reduced *Hes1* expression, precedes RA-stimulated differentiation of β-cells.

### Sequential roles for RA in the generation of β-cells

**Figure 4:**
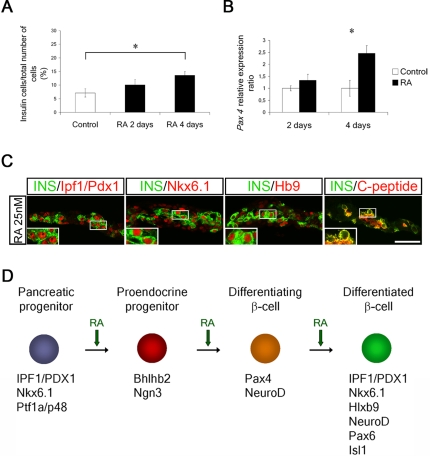
Temporal changes in the expression of Bhlh and homeobox genes in response to RA. (A) Quantification of Ins^+^ cells in control pancreatic explants (n =  6) and pancreatic explants exposed to 25 nM RA for the first 2 (n = 3) or 4 days (n = 4) of the 6 day in vitro culture period displayed as % positive cells/total number of cells. Data show mean±S.E.M, *p<0.05 **p<0.01. (B) qRT-PCR using *Pax4* specific primers on cDNA from control and RA treated e10.5 pancreatic explants cultured for two (n = 13) or four days (n = 9). Data represent the mean value±S.E.M. *p<0.05, **p<0.01. (C) 6d RA treated explants double-stained for insulin (green) and Ipf1/Pdx1, Nkx6.1, Hb9 or C-peptide (all red). (D) Schematic model summarizing the multiple proposed roles of RA during pancreatic development. Our data provides evidence that RA sequentially specifies: i) proendocrine cells; ii) committed pre-β-cells; and finally iii) differentiated β-cells. Scale bar = 30 µM in (C) (7,5 µM for the enlarged inserts).

The induction of Ngn3^+^ endocrine progenitor cells and the preferential generation of β-cells by RA, suggests that RA may promote the generation of a set of Ngn3^+^ cells that consequently are destined to generate β-cells. Alternatively, similar to its role during motor neuron differentiation [9]–[11], RA may have distinct sequential functions during the generation of β-cells, i.e. inducing both Ngn3 expression and the further differentiation of Ngn3^+^ cells into β-cells. To distinguish between these two possibilities, e10.5 pancreatic buds were cultured in presence of RA for only the first 2 or 4 days of the total 6 day culture period. No significant increase in the number of Ins^+^ cells was observed in explants exposed to RA for only the first 2 of the total 6 day culture period (Fig. 4a). In explants exposed to RA for the first 4 of the total 6 days, there was a significant increase in the number of Ins^+^ cells (Fig. 4a), though smaller than that observed in explants exposed to RA the entire 6 day culture period (Fig. 2b and data not shown). Thus, RA needs to be added throughout the culture period to ensure Ins^+^ cell differentiation.

Like the sequential expression of Bhlh genes (Fig. 3c), homeobox genes linked to β-cell differentiation were expressed in a sequential manner during RA-stimulated Ins^+^ cell differentiation. The expression of *Pax4*, a critical factor in specification of β-cell identity [25], was not significantly increased after 2 days of RA exposure but a ∼2.5 fold increase in *Pax4* expression was observed after 4 days of RA exposure (Fig. 4a). The Ins^+^ cells induced by RA expressed the additional β-cell markers Ipf1/Pdx1, Nkx6.1 and Hb9, as well as C-peptide and Glut2, after 6 days of RA exposure (Fig. 4b and data not shown). RA therefore mediates sequential induction of the Bhlh transcription factors *Ngn3, Bhlhb2,* and *NeuroD* that promote the generation of endocrine cells, followed by induction of the homeobox gene, *Pax4*, that determines β-cell identity, and finally the generation of insulin producing β-cells that express the homeodomain proteins Ipf1/Pdx1, Nkx6.1 and Hb9 (Fig. 4c). Taken together these results provide evidence that, in analogy with the generation of motor neurons in the spinal cord, RA acts at multiple levels in the generation of β-cells in the pancreas.

## Discussion

In the developing mouse pancreas, β-cells increase in large numbers from ∼e14.5 onwards, suggesting that intrinsic and/or extrinsic signals favour β-cell differentiation at these stages. Transcription factors that control specification of pancreatic endocrine progenitor cells and their further differentiation into β-cells have been identified [13]. In contrast, secreted signals that may promote the large increase in generation of β-cells remain to be identified. In this study, we show that the RA synthesizing enzyme *Raldh1* is expressed in the developing mouse and human pancreas at stages when β-cells are predominantly generated. Moreover, using an explant assay of pancreatic endocrine cell differentiation, we provide evidence that RA activates a program of cell differentiation that recapitulates the normal temporal program of β-cell differentiation.

In *Raldh2* mutant mice, Ipf1/Pdx1 expression is lost in the dorsal but not ventral pancreatic bud and mutant mice present with dorsal but not ventral pancreatic agenesis [17], [18]. However, the dorsal but not ventral pancreatic mesenchyme fails to form in *Raldh2* mutants [17], suggesting that this phenotype is indirect. Consistent with this possibility early pancreatic markers such as Hb9 and Isl1 are expressed in e9.5 *Raldh2* mutant dorsal pancreatic endoderm [17]. Moreover, maternal RA administration restored the formation of dorsal pancreatic mesenchyme, Ipf1/Pdx1 expression and thus pancreatic development in *Raldh2* mutant embryos [17]. Loss of dorsal pancreatic mesenchyme and Ipf1/Pdx1 expression are also observed in *Isl1* [26] and *N-cad* [27] mutant embryos, and dorsal pancreatic development could be rescued in both mutants by co-culturing the pancreatic endoderm with wild-type mesenchyme [26], [27]. In *Xenopus* and zebrafish, induction of pancreatic development is inhibited following treatment of early, pre-pancreatic embryos, with the RA-antagonist BMS493, and embryos treated with exogenous RA prior to the initiation of the pancreatic development show ectopic expression of pancreatic markers such as insulin [28]–[31]. Collectively, these data provide evidence that RA is required during early (dorsal) pancreas development, at least in part by promoting the development of dorsal pancreatic mesenchyme. In addition, the apancreatic phenotype of *Ipf1/RAR403* tg mice suggests a direct role for RA signaling in both the early dorsal and ventral pancreatic epithelium after the onset of Ipf1/Pdx1 expression. The lack of pancreas in these mice unfortunately precluded the establishment of a transgenic line that would allow detailed analysis of the pancreatic developmental defect.

Differentiation of all pancreatic endocrine cells requires the transient activity of the proendocrine gene *Ngn3* in progenitor cells [1]–[6]. Different classes of endocrine cells are generated at different stages of embryonic development of the pancreas and forced expression of *Ngn3* in early pancreatic progenitor cells generates predominantly glucagon-producing cells whereas later induction generates β-cells and other endocrine cell types [1], [2], [5]. Our data show that the temporal expression of the RA-synthesizing enzyme *Raldh1* in the developing pancreas coincides with the large increase in β-cells from ∼e14.5 and onwards. Using an explant assay, we also show that RA and RA precursors selectively stimulate β-cell differentiation, resulting in both a relative and absolute increase in β-cell numbers. *Raldh1* mutant mice have been generated and are viable, suggesting that they are not devoid of β-cells [32]. Whether this reflects increased expression of any of the other *Raldhs* in the pancreas of *Raldh1* mutant mice at the time of β-cell differentiation will have to await future analyses of *Raldh2-4* expression in *Raldh1^−/−^* mice. An alternative, RALDH-independent mode of RA synthesis mediated by Cyp1b1 was recently reported [33] and qRT-PCR analyses detected low level expression of *Cyp1b1* in mouse e13 and e15 pancreas and in 8–19 weeks human fetal pancreas (data not shown).

By using medium containing high levels of serum that favors growth of explants, previous studies suggest that RA antagonizes growth and exocrine cell differentiation leading to a relative but not absolute increase of β-cells [34], [35]. In the developing pancreas, Fibroblast growth factor (FGF) signals provided by the mesenchyme stimulate growth and differentiation of the exocrine pancreas [30], [36], [37]. RA and FGFs are known to exert opponent activities [38] and under culture conditions promoting growth, RA is likely to primarily antagonize the stimulatory effect of FGFs on growth and differentiation of the exocrine pancreas. In support of this, FGF10 was shown to antagonize the negative effects of RA on pancreatic growth, morphogenesis, and exocrine differentiation [34]. β-cell differentiation in the mouse embryo becomes prominent when the mesenchyme/epithelium ratio, and thus growth decreases [7], [8]. This phase, sometimes referred to as the secondary transition [7], coincides temporally with *Raldh1* expression in the pancreas. Our culture conditions were designed to mimic this phase of β-cell differentiation by using defined, minimal media without serum, which allows survival but not growth of the pancreatic explants. Under these conditions, RA or RA precursors selectively stimulate differentiation of pancreatic progenitors into β-cells.

Together, our results provide strong evidence that RA promotes both the generation of Ngn3^+^ endocrine progenitor cells and their further differentiation into β-cells. The increase in Ngn3^+^ cells is evident already after 2 days of RA exposure although *Hes1* expression is significantly reduced first after 4 days of RA exposure, leaving open the possibility that the increase in Ngn3^+^ cells is independent of *Hes1* expression. Whether the early increase in *Bhlhb2* expression already at day 2 of RA exposure, directly or indirectly, promotes a parallel increase in Ngn3 expression, similar to the positive effects of Olig2 on Ngn2 expression [9.11], will require further analyses. Nevertheless, the early increase in the expression of *Bhlhb2,* which has Notch antagonizing activity [24], is in agreement with the subsequent reduced expression of *Hes1* observed at day 4 of RA exposure. The identification of RA as a secreted signal that promotes the selective generation of β-cells, and increased availability of human ES cell lines that respond to experimental protocols that allow efficient and reproducible generation of pancreatic endoderm, will help to develop stem cell-based β-cell replacement therapy.

## Materials and Methods

### Human fetal pancreas isolation

The fetal pancreatic studies were approved by the Human Subject Research Office, Miami, Florida, in compliance with US legislation and the guidelines of our institution. Fetal pancreatic tissue was obtained following written maternal consent. Elective termination of pregnancy was performed by aspiration between 8 and 19 weeks of development. Warm ischemia lasted less than 30 minutes.

### Animals and generation of transgenic mice

Animal studies were approved by the Institutional Animal Care and Use Committee of Umeå University and conducted in accordance with the Guidelines for the care and use of Laboratory Animals. Human RARα403 was subcloned behind the *Ipf1/Pdx1* promoter and the *RIP* promoter respectively. Transgenic mice were generated as described^16^.

### Immunohistochemistry and *in situ* hybridization

Immunohistochemical analyses were carried out as described elsewhere^16^. Primary antibodies used were**:** rabbit anti-Ipf1^39^ (Ohlsson et al., 1993), rabbit anti-Ngn3^40^, guinea pig anti-Insulin (Linco), rabbit anti C-peptide (Linco), rabbit anti-Glucagon (Linco), rabbit anti-Nkx6.1 (raised against a GST-Nkx6.1 fusion protein by AgriSera AB, Vännäs, Sweden) and rabbit anti-Hb9 (kindly provided by T. M. Jessell). Secondary antibodies were: Cy3 anti-rabbit (Jackson), Alexa Fluor 488 anti-guinea pig and Alexa Fluor 594 anti-rabbit (Molecular Probes). *In situ* hybridizations were performed as previously described^16^. Images were acquired using a Zeiss Axioplan imaging microscope or a Nikon confocal microscope C1 fitted with an Ar, He/Ne and a blue diode laser. Digitalized confocal images were assigned red and green pseudocolors for Cy3 and Alexa Fluor 488 respectively.

### *In vitro* cultures

Isolated e10.5 dorsal pancreatic buds were cultured on filters (Millipore) in 24-well plates (Costar) with 400 µl of medium consisting of DMEM-glutamax1 (Gibco), 1×N_2_-supplement (Gibco) and pen/strep (Gibco), and different concentrations of *all trans* retinoic acid or retinol (Sigma). Explants were cultured for up to six days in 37°C and 5% CO_2_ and medium was changed every other day. After culture, explants were washed twice in PBS, fixated in 4% PFA at 4°C for 20 minutes followed by two more washes in PBS. Explants were frozen in drops of Tissue Tek OCT applied on glass slides (Menzel) and stored at −80°C until sectioned (8 µm sections) and analyzed by immunohistochemistry.

### Quantification of mRNA expression levels

cDNA was prepared from explants, fetal mouse and human pancreas and qRT-PCR analysis of the cDNA was performed essentially as described^41^. Expression of TATA box binding protein (TBP) and human 18S ribosomal RNA (18S) was used to normalize expression levels. Primer sequences are found in Supplementary Table S1.

### Statistical analysis

All data is presented as mean value±standard errors (S.E.M.). All statistical analyses were performed using the two-tailed, two sample Student́s t-test and P values <0.05 were considered as significant.

## Supporting Information

Figure S1 RALDH1 and 2 expression in mouse embryos
(1.37 MB TIF)

Figure S2 Bhlhb2 and Ngn3 expression in e13.5 mouse pancreas.
(6.05 MB TIF)

Table S1 Primer sequences.
(0.04 MB DOC)
