# Supplementary figures and images for: Retinoic Acid Promotes the Generation of Pancreatic Endocrine Progenitor Cells and Their Further Differentiation into β-Cells
Source: PLoS One. 2008 Jul 30;3(7):e2841. doi: 10.1371/journal.pone.0002841 (PMC2475501; doi:10.1371/journal.pone.0002841)

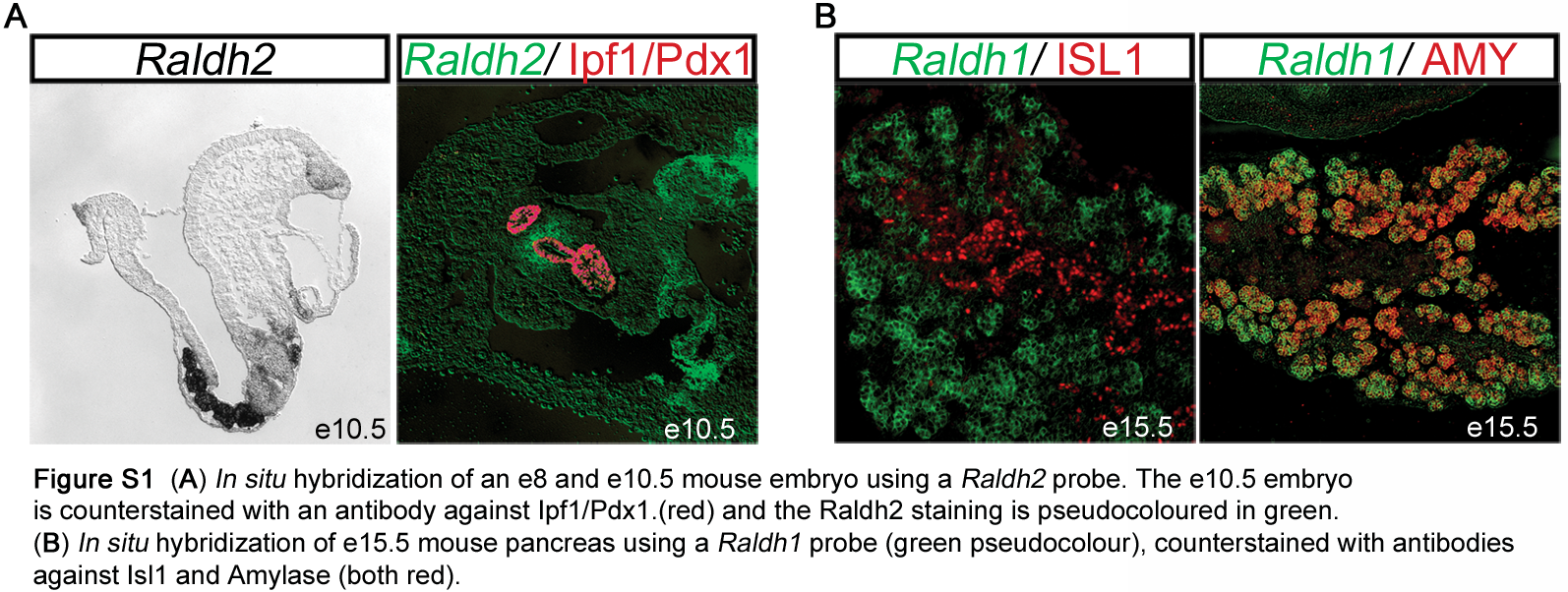

Supplement: Figure S1 — RALDH1 and 2 expression in mouse embryos (1.37 MB TIF) [file pone.0002841.s001.tif]

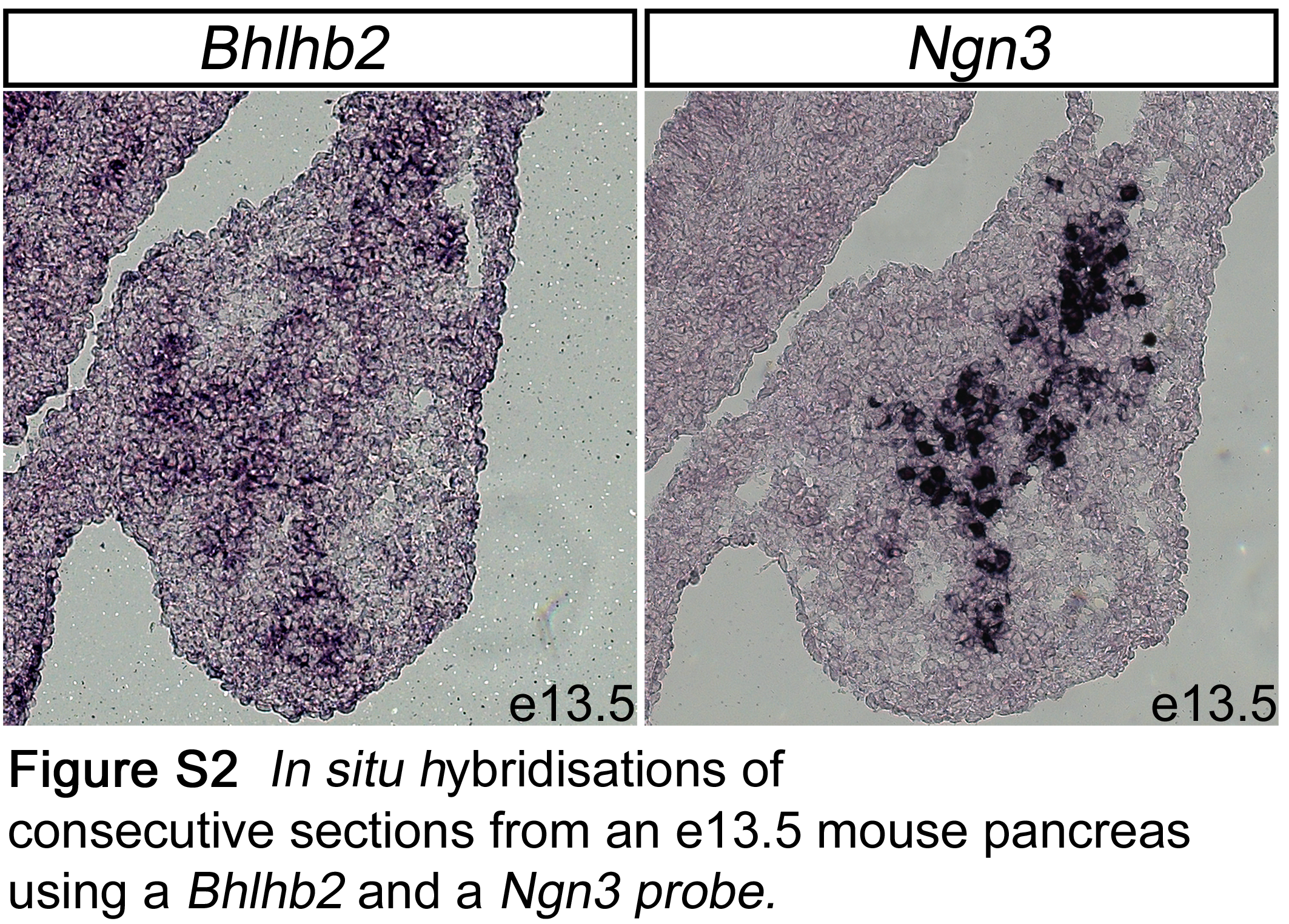

Supplement: Figure S2 — Bhlhb2 and Ngn3 expression in e13.5 mouse pancreas. (6.05 MB TIF) [file pone.0002841.s002.tif]
