# Supplementary material for: Retinoic Acid Promotes the Generation of Pancreatic Endocrine Progenitor Cells and Their Further Differentiation into β-Cells
Source: PLoS One. 2008 Jul 30;3(7):e2841. doi: 10.1371/journal.pone.0002841 (PMC2475501; doi:10.1371/journal.pone.0002841)
Supplement: Table S1 — Primer sequences. (0.04 MB DOC) [file pone.0002841.s003.doc]

| **Supplemental Data table 1** | | |
| --- | --- | --- |
| **Genotyping primers** | Forward | Reverse |
| Ipf1/RARα403 and Ipf1/VP16RAR | 5'-GGGAAGAGGAGATGTAGACTT-3' | 5'-AGCAAGGCTTGTAGATGC-3' |
| Rip/RARα403 and Rip/VP16RAR | 5'-GGTGCTTTGGACTATAAAGC-3' | 5'-AGCAAGGCTTGTAGATGC-3' |
| **Mouse real-time PCR primer sequences:** |  |  |
| Gene | Forward | Reverse |
| *TBP* | 5’-GAATTGTACCGCAGCTTCAAAA-3’ | 5’- AGTGCAATGGTCTTTAGGTCAAGTT-3’ |
| *Bhlhb2* | 5’-TGGTTCTGGAGCTTACGTTGAAG-3’ | 5’-TGCAGGGCAATGATTTTCTG-3’ |
| *Hes1* | 5’-GTCTAAGCCAACTGAAAACACTGATT-3’ | 5’-TGCCTTCTCTAGCTTGGAATGC-3’ |
| *NeuroD* | 5’-AGGCAGCCCTTTTGCTAAGAT-3’ | 5’-TTCCAAAGGCAGTAACGACAATAA-3’ |
| *Pax4* | 5’-ACCTCATCCCAGGCCTATCTC-3’ | 5’-TGAGGAGGAAGCCACAGGAA-3’ |
| **Human real-time PCR primer sequences:** |  |  |
| *18S* | (ref. # Hs99999901_s1) |  |
| *RALDH1* | (ref. # Hs00167445-m1) |  |
| *RALDH2* | (ref. # Hs00180254-m1) |  |
| *RALDH3* | (ref.# Hs00167476-m1) |  |
| *RALDH12* | (ref.# Hs00164383-m1) |  |
| *INS* | (Hs02741908-m1) |  |
